# Exploring Facial Index as an Indicator of Ethnic Lineage in Upper Himalayan Indigenous Tribal Populations

**DOI:** 10.7759/cureus.84773

**Published:** 2025-05-25

**Authors:** Dipen Dabhi, Yatiraj Singi, Nirmal Nagar, Jasmine Jain, Varun Modgil, Reshma Rathore, Diksha Chhabra, Sanchit Jain, Amit Jangid, S N Krishnagopal

**Affiliations:** 1 Forensic Medicine and Toxicology, All India Institute of Medical Sciences, Bilaspur, Bilaspur, IND; 2 Forensic Medicine and Toxicology, Dayanand Medical College and Hospital, Ludhiana, IND; 3 Forensic Medicine and Toxicology, All India Institute of Medical Sciences, Bhopal, Bhopal, IND; 4 Forensic Medicine and Toxicology, National Capital Region Institute of Medical Sciences, Meerut, IND; 5 Forensic Medicine and Toxicology, All India Institute of Medical Sciences, Rishikesh, Rishikesh, IND

**Keywords:** ethnic group, facial index, identification, indigenous, prosopic index, race, tribal population

## Abstract

Background: Facial features differ widely among individuals and populations, shaped by a mix of biological and environmental influences. These differences can be observed between groups from various regions and even within communities living in the same area. The Facial Index (FI) is commonly used to compare facial proportions across different populations.

Aim and objectives: This study aims to estimate the FI, classify facial types based on FI, evaluate sexual dimorphism, and generate reference data on facial morphology. The study also aimed to compare FI values with those of other populations.

Materials and methods: A total of 413 individuals participated in the study, comprising 247 (59.8%) male participants and 166 (40.2%) female participants, aged 18-50 years, from the tribal districts of Kinnaur, Lahaul, and Spiti. Facial measurements were recorded using standard anthropometric tools, and the FI was calculated. Data were analyzed using IBM SPSS Statistics software version 27 (IBM Corp., Armonk, NY).

Results: The mean values of morphognomic facial length (MFL), bizygomatic breadth (BZB), and FI were 130.69 ± 8.09 mm, 112.81 ± 9.81 mm, and 116.42 + 9.31, respectively. Male participants exhibited significantly greater facial dimensions in terms of both MFL and BZB compared to female participants, but there was no significant difference in FI. The hyperleptoprosopic facial type was predominant, observed in 386 (93.5%) individuals, followed by 20 (4.8%) leptoprosopic, six (1.5%) mesoprosopic, and one (0.2%) euryprosopic types. Among male participants, 233 (94.3%) were hyperleptoprosopic compared to 153 (92.2%) female participants. The FI did not show a significant correlation with age, height, weight, or body mass index (BMI). However, MFL exhibited weak to moderate positive correlations with age, height, weight, and BMI. BZB also showed weak but statistically significant positive correlations with height and weight.

Conclusion: The hyperleptoprosopic facial type was the most common in both male and female participants, with the FI showing no significant differences between sexes. These findings enhance the anthropometric characterization of this indigenous population, providing important reference data that can be applied in forensic investigations, anthropological studies, and clinical assessments.

## Introduction

The Facial Index (FI) is a key anthropometric parameter used in physical anthropology to delineate similarities and distinctions among various ethnic and racial groups. It provides valuable information on craniofacial structure and variation [1]. Facial morphology is influenced by a range of factors, including genetic inheritance, sex, age, geographic location, and environmental conditions such as climate [2]. Although environmental adaptation has shaped facial traits over generations, genetic inheritance remains the predominant determinant of craniofacial characteristics [3]. These features not only vary significantly across different racial populations but also demonstrate considerable variation within the same racial group across diverse geographic regions, as well as among distinct ethnic communities residing in the same area [4].

The indigenous populations of the tribal districts of Himachal Pradesh, namely, Kinnaur, Lahaul, and Spiti, are believed to be descendants of ancient immigrants from the Tibetan region [5,6]. These communities have continued to reside in this area for generations. The present study was conducted to assess the FI among the indigenous population of these districts, to classify individuals into different facial types, and to examine sex-based differences. It is the first study to evaluate the FI in this uniquely isolated region of the upper Himalayas and aims to establish baseline data for future research in the fields of physical anthropology, forensic medicine, maxillofacial surgery, and plastic surgery concerning this population.

## Materials and methods

This cross-sectional study was conducted between August 2023 and August 2024 among adults residing in the tribal districts of Kinnaur, Lahaul, and Spiti in Himachal Pradesh, India. Ethical approval was obtained from the Institutional Ethics Committee (Biomedical and Health Research) at All India Institute of Medical Sciences, Bilaspur (reference number: 68/23, dated 05/08/2023).

Participants were selected from families that had resided in these tribal districts for at least three generations to ensure representation of inherited traits of the native tribal population. Prior to initiating the study, permission was obtained from the head of each village, and written informed consent was secured from all participants.

According to the 2011 Census, the combined population of the tribal districts is 115,865 [7]. The required sample size was calculated using the formula:

\[n = \frac{Z^2 \cdot p \cdot (1 - p)}{e^2}\]

where n = required sample size, Z = Z-score for a 95% confidence level (1.96), p = expected prevalence (assumed 0.5 for maximum sample size), and e = margin of error (0.05).

This yielded a sample size of 383, which corresponds to 0.33% of the total population. A total of 413 individuals were eventually included in the study. Participants aged 18-50 years were chosen as this age range represents the adult population, by which time the development and maturation of facial bones is generally complete. This ensures that the facial dimensions measured reflect stable anatomical characteristics, thereby reducing variability due to ongoing growth or age-related skeletal changes. The upper limit of 50 years was set to exclude changes in facial parameters due to osteoporosis. Participants were selected using cluster sampling via a door-to-door survey. Individuals with visible deformities of the head, face, or vertebrae, or with a history of craniofacial trauma, surgery, or developmental or metabolic disorders, were excluded.

To minimize inter-observer bias, all anthropometric measurements were conducted by a single observer using standard instruments, following the method described by Vallois [8]. Each measurement was taken three times during the same sitting, and the average of the three readings was used for analysis. Participants were seated in a relaxed posture with the head aligned anatomically in the orbito-ocular (Frankfurt horizontal) plane.

Morphognomic facial length (MFL) was defined as the vertical distance between the nasion (the midpoint of the nasofrontal suture) and the gnathion (the lowest point on the lower border of the chin at the midline). A standard straight vernier calliper was used, with the fixed tip placed at the gnathion and the movable tip adjusted to touch the nasion.

Bizygomatic breadth (BZB), or the widest horizontal facial breadth, was measured between the two zygomatic prominences (the most lateral points of the zygomatic arches). A standard spreading calliper was used for this purpose. The zygomatic prominences were located through palpation, and the calliper ends were gently positioned on these landmarks to feel the underlying bone. Minor adjustments were made to locate the maximum width, which was recorded.

Statistical analysis

FI was calculated using the following formula: \[ FI = \left( \frac{MFL}{BZB} \right) \times 100 \]

Facial types based on FI are classified as follows: hypereuryprosopic (FI < 79.9), euryprosopic (FI = 80.0-84.9), mesoprosopic (FI = 85.0-89.9), leptoprosopic (FI = 90.0-94.9), and hyperleptoprosopic (FI > 95) [2]. Data analysis was conducted using IBM SPSS Statistics software version 27 (IBM Corp., Armonk, NY). Descriptive statistics (mean and standard deviation (SD)) were used for all continuous variables. The Shapiro-Wilk test was used to assess the normality of data distribution. For gender comparisons, the independent samples t-test was applied to normally distributed variables, and the Mann-Whitney U test was used for non-normally distributed variables. Pearson's and Spearman's correlation coefficients were used to evaluate the relationships between facial measurements (FI, MFL, and BZB) and demographic or anthropometric parameters (age, height, weight, and body mass index (BMI)), depending on the distribution of the variables. A p-value < 0.05 was considered statistically significant. The primary outcome variable was the FI, and the key explanatory variables included MFL, BZB, sex, age, height, weight, and BMI.

## Results

A total of 413 individuals participated in the study, comprising 247 (59.8%) male participants and 166 (40.2%) female participants. The mean age of the participants was 37.08 ± 8.97 years. The average weight and height were 63.21 ± 12.15 kg and 162.81 ± 13.96 cm, respectively. The mean values and standard deviations (SD) for FI, MFL, and BZB were 116.42 ± 9.31, 130.69 ± 8.09 mm, and 112.81 ± 9.81 mm, respectively. These findings represent the general craniofacial and physical characteristics of the studied population (Table 1).

**Table 1:** Descriptive statistics of study participants Values are presented as mean ± SD. Age, weight, and height represent general anthropometric characteristics. SD: standard deviation, MFL: morphognomic facial length, BZB: bizygomatic breadth, FI: Facial Index

Parameter	Mean ± SD
Age (years)	37.08 ± 8.97
Weight (kg)	63.21 ± 12.15
Height (cm)	162.81 ± 13.96
MFL (mm)	130.69 ± 8.09
BZB (mm)	112.81 ± 9.81
FI	116.42 + 9.31

Facial parameters were compared between male and female participants to assess statistically significant gender differences. The independent t-test was applied to variables with normal distribution, while the Mann-Whitney U test was used for those not following a normal distribution. Male participants exhibited significantly greater facial dimensions in terms of both MFL and BZB compared to female participants, but there was no significant difference in FI. These findings indicate the absence of sexual dimorphism in the FI of the studied population, with male participants generally having longer and broader faces than female participants (Table 2).

**Table 2:** Comparison of morphometric facial parameters between male and female participants Values are presented as mean ± SD. Statistical significance was assessed using the independent samples t-test for normally distributed variables and the Mann-Whitney U test for non-normally distributed variables. A p-value < 0.05 was considered statistically significant. SD: standard deviation, MFL: morphognomic facial length, BZB: bizygomatic breadth, FI: Facial Index

Parameters	Male participants (mean ± SD)	Female participants (mean ± SD)	Test	P-value
MFL (mm)	133.91 ± 7.24	125.9 ± 6.83	T-test, t = 11.407	<0.001
BZB (mm)	115.2 ± 10.03	109.27 ± 8.3	Mann-Whitney U test, W = 27980	<0.001
FI	116.87 ± 9.4	115.75 ± 9.17	T-test, t = 1.207	0.228

A total of 386 (93.5%) individuals exhibited a hyperleptoprosopic facial index, followed by 20 (4.8%) leptoprosopic, six (1.5%) mesoprosopic, and one (0.2%) euryprosopic types. Among male participants, 233 (94.3%) were hyperleptoprosopic, 11 (4.5%) leptoprosopic, and three (1.2%) mesoprosopic. Among female participants, 153 (92.2%) were hyperleptoprosopic, nine (5.4%) leptoprosopic, three (1.8%) mesoprosopic, and one (0.6%) euryprosopic.

The correlation between FI, MFL, BZB, and various body measurements (age, height, weight, and BMI) was analyzed. The FI showed no significant correlation with age, height, weight, or BMI, indicating that the FI is largely independent of these factors. In contrast, MFL exhibited a weak but significant positive correlation with age and moderate positive correlations with height and weight. Additionally, MFL showed a weak positive correlation with BMI. BZB demonstrated weak but significant positive correlations with height and weight, but no significant correlation with age (Table 3).

**Table 3:** Correlation of FI, MFL, and BZB with age, height, weight, and BMI Correlation strength was interpreted as weak (|r| < 0.3), moderate (0.3 ≤ |r| < 0.5), or strong (|r| ≥ 0.5). A p-value < 0.05 was considered statistically significant. FI: Facial Index, MFL: morphognomic facial length, BZB: bizygomatic breadth, BMI: body mass index, r/ρ: Pearson's/Spearman's correlation coefficient

Variable pair	Correlation coefficient (r/ρ)	P-value	Interpretation
FI and age	ρ = 0.05	0.340	Not significant
FI and height	ρ = 0.03	0.535	Not significant
FI and weight	r = 0.01	0.899	Not significant
FI and BMI	ρ = -0.01	0.821	Not significant
Age and MFL	ρ = 0.11	0.024	Weak, significant
MFL and height	ρ = 0.38	<0.001	Moderate, significant
MFL and weight	r = 0.37	<0.001	Moderate, significant
MFL and BMI	ρ = 0.19	<0.001	Weak, significant
Age and BZB	ρ = 0.03	0.539	Not significant
BZB and height	ρ = 0.25	<0.001	Weak, significant
BZB and weight	ρ = 0.27	<0.001	Weak, significant

## Discussion

The present study revealed that the majority (93.5%) of individuals from the tribal populations of Kinnaur, Lahaul, and Spiti in the upper Himalayas exhibited a hyperleptoprosopic facial type, characterized by long and narrow faces. This finding differs notably from observations in other studies on Tibetan populations residing in Tibet and other regions. One study reported a predominance of hypereuryprosopic types among Tibetans, with 45.6% of male participants and 25.5% of female participants displaying this facial form [2]. Similarly, a study on Mongolian youth in Inner Mongolia found high frequencies of euryprosopic (46.67% in male participants) and hypereuryprosopic (56.67% in female participants) types [9]. Data from Tibetan populations living in Rajasthan, India, also contrast with the present results. Fulwaria et al. reported that 70% were euryprosopic and 25% mesoprosopic, with no hypereuryprosopic types noted [10]. These differences may be attributable to genetic admixture or adaptation to Rajasthan's lower altitude environment, in contrast to the high-altitude Himalayan setting of this study.

East Asian populations show further variability in facial morphology. Among Han Chinese, individuals from northern China are more often hyperleptoprosopic (32.3% in men and 37.0% in women), whereas those from southern China display a higher proportion of euryprosopic (26.0% in men) and leptoprosopic (25.9% in women) types [11]. These trends are more consistent with the present findings than with Tibetan group data, suggesting a possible craniofacial affinity between the upper Himalayan tribal population and northern East Asians.

The current study's results also align with findings from central India. A study in Madhya Pradesh recorded a predominantly hyperleptoprosopic facial type (79.5%), with no cases of euryprosopic or hypereuryprosopic types [12]. In contrast, populations from northern India are marked by higher proportions of euryprosopic (53.2%) and mesoprosopic (21.6%) types [13]. Northeastern Indian populations, such as those in Manipur, display a more heterogenous distribution: mesoprosopic (30.1%), leptoprosopic (30.1%), hyperleptoprosopic (14.6%), and hypereuryprosopic (7.7%) types [14]. These regional differences may reflect both genetic heterogeneity and ecological adaptation across India's diverse environments [12-14].

Among Nepalese populations, the mesoprosopic facial type is most common (38.7%), followed by euryprosopic (29.3%) and leptoprosopic (23.3%), with hyperleptoprosopic forms being rare (0.3%) [15]. Despite Nepal's geographical proximity to Himachal Pradesh, facial morphology among Nepalese differs notably, suggesting that genetic lineage may exert greater influence than geographical closeness alone.

From a genetic perspective, craniofacial distinctions among populations may reflect divergent evolutionary histories. Genetic studies have shown affinities between Nepalese, northeastern Indians, and Tibetans through the Tibeto-Burman branch of the Sino-Tibetan language family [16,17]. Even within these broadly related groups, ecological and evolutionary pressures, including altitude, solar radiation, and diet, likely contribute to morphological divergence. Buretic-Tomljanovic et al. proposed an inverse association between sunshine duration and the morphological facial index, which may help explain the wider faces observed in Tibet, where solar radiation is intense [18,19]. Clustering analyses indicate that Tibetans are genetically and morphometrically closer to northern Asian groups such as Mongols, Daur, and northern Han Chinese [20]. The upper Himalayan tribal populations studied here may represent a genetically distinct group shaped by long-term ecological isolation and environmental pressures associated with high-altitude life.

When compared with other Indian populations, a study from North India also reported hyperleptoprosopic types in both men and women [21]. Research in Himachal Pradesh identified a leptoprosopic pattern among men and a mesoprosopic pattern among women [22], while another study found hypereuryprosopic types in men [23]. Other studies from Northern India describe facial types ranging from hypereuryprosopic to mesoprosopic [24-26]. In Southern India, reports commonly document euryprosopic [27] and mesoprosopic [28] types in both sexes, with men showing higher FI values than women.

The FI values observed in this study are substantially higher than those reported from most other Indian regions. This suggests a distinctly hyperleptoprosopic morphology in this high-altitude tribal population, potentially resulting from an ancestral Tibetan lineage combined with environmental and genetic influences specific to the Himalayas. Collectively, these findings provide important insights into India's anthropometric variation and demonstrate the relevance of both genetic and ecological factors in shaping human craniofacial characteristics.

Limitations

This study was conducted on a specific population that is unique due to its geographical location and inherited ethnic lineage. Therefore, the findings and calculated data may primarily be applicable to this particular localized group or populations with similar facial index characteristics and demographic features.

## Conclusions

This study provides valuable data on the craniofacial characteristics of indigenous tribal populations residing in the high-altitude regions of Himachal Pradesh. The facial morphology observed reflects distinct ethnic and regional traits likely influenced by both genetic and environmental factors. While regional variation was evident, no significant gender-based differences in facial dimensions were found. These observations contribute to understanding human adaptation in extreme environments and may serve as a reference for future research in forensic anthropology, population studies, and human biology.
